# Lifestyle Modulators of Neuroplasticity: How Physical Activity, Mental Engagement, and Diet Promote Cognitive Health during Aging

**DOI:** 10.1155/2017/3589271

**Published:** 2017-06-12

**Authors:** Cristy Phillips

**Affiliations:** Department of Physical Therapy, AState, Jonesboro, AR, USA

## Abstract

The number of the elderly across the globe will approximate 2.1 billion by 2050. Juxtaposed against this burgeoning segment of the population is evidence that nonpathological aging is associated with an increased risk for cognitive decline in a variety of domains, changes that can cause mild disability even before the onset of dementia. Given that pharmacological treatments that mitigate dementia are still outstanding, alternative therapeutic options are being investigated increasingly. The results from translational studies have shown that modifiable lifestyle factors—including physical activity, cognitive engagement, and diet—are a key strategy for maintaining brain health during aging. Indeed, a multiplicity of studies has demonstrated relationships between lifestyle factors, brain structure and function, and cognitive function in aging adults. For example, physical activity and diet modulate common neuroplasticity substrates (neurotrophic signaling, neurogenesis, inflammation, stress response, and antioxidant defense) in the brain whereas cognitive engagement enhances brain and cognitive reserve. The aims of this review are to evaluate the relationship between modifiable lifestyle factors, neuroplasticity, and optimal brain health during aging; to identify putative mechanisms that contribute positive brain aging; and to highlight future directions for scientists and clinicians. Undoubtedly, the translation of cutting-edge knowledge derived from the field of cognitive neuroscience will advance our understanding and enhance clinical treatment interventions as we endeavor to promote brain health during aging.

## 1. Introduction

The number of elderly across the globe will approximate 2.1 billion by the year 2050 [1, 2]. Accompanying this increase will be the personal, social, and economic burden of care for individuals with age-related disorders. These challenges are even more worrisome given that nonpathological aging is associated with decrements in key regions of the brain vital for cognitive function and, thereby, decline in several cognitive domains (including memory, attention, speed of processing, and executive function) [3, 4], changes that may result in mild disability even prior to the onset of dementia. Notwithstanding, pharmacological treatments that mitigate dementia are still outstanding, creating an imperative to diversify efforts to find efficacious alternatives. Modifiable lifestyle factors are among the candidate therapeutics particularly well-poised to mitigate age-related disorders [5–11]. Evidence strongly suggests that the maintenance of adequate levels of physical activity (PA), engagement in cognitive stimulation, and optimization of nutritional intake can increase neural plasticity and resilience of the brain [12–15].

The ability of neurons in the brain to change and reorganize continuously to meet the dynamic demands of the internal and external environment is termed neuronal plasticity. This process is dependent on membrane depolarization of the neuron, stimulus-induced synaptic activity, and subsequent changes in dendritic morphology, central hallmarks of learning and memory. Importantly, long-term PA moderates processes that are cornerstone for neuroplasticity [16]. Van Praag et al. demonstrated that mice that were given voluntary access to running wheels exhibited selective enhancement of long-term potentiation (LTP) in the dentate gyrus [17], a phenomenon linked with concomitant increases in brain-derived neurotrophic factor (BDNF) [18]. Eadie et al. demonstrated that long-term PA significantly increased dendritic length, dendritic complexity, and spine density in the dentate gyrus of mice [19]. Stranahan et al. demonstrated that long-term voluntary wheel running in rats induced changes in spine density along with changes in arborization and spine morphology [20]. Altogether, these findings suggest that PA induces neuroplastic changes in brain structure and function and, therefore, may be an effective component of therapeutic regimes that aim to improve cognition. Interestingly, other work suggests that mental engagement and dietary factors also effectuate changes in plasticity by altering neurotrophic signaling, neurogenesis, inflammation, stress response, and antioxidant defense mechanisms, which are outcomes similar to those implicated in the cognitive response to PA [16].

Knowledge of the dynamic relationship between brain plasticity and lifestyle factors creates an imperative to better understand and harness these links to promote healthy aging and forestall the onset of disease. Several national bodies have affirmed this notion, including the National Institutes of Health [21], the Centers for Disease Control, the Alzheimer's Association, and the American Association of Retired Persons (AARP). Accordingly, the purpose of this review is to (1) explicate key lifestyle factors (in particular PA, cognitive engagement, and diet) that can be harnessed to enhance neuroplasticity and optimal brain health; (2) explore the putative mechanisms by which these factors affect age-related biology; and (3) highlight implications for clinicians and researchers.

## 2. Physical Activity

Numerous studies have reported a robust relationship between higher levels of PA and improved learning and memory [22, 23]. Epidemiological studies show that regular PA reduces the risk of cognitive decline in aging adults [15, 24–26], with some evidence intimating that midlife PA may be especially beneficial. A population-based study of PA at midlife, followed up 26 years later with an assessment of late-life cognitive function, found that groups who participated in PA during midlife exhibited a faster speed of processing along with better memory and executive function. Additionally, those in the moderate PA group were significantly less likely to have dementia in late life [15]. A meta-analysis of 29 randomized controlled trials (*n* = 2049) showed that aerobic exercisers exhibited improvements in attention, processing speed, memory, and executive function [27]. Another meta-analysis of 15 prospective studies (*n* = 33,816 persons without dementia) reported that PA consistently resulted in a protective effect at all levels of activity [28]. Findings from a study of school children clearly demonstrates a positive correlation between PA and academic performance [29]. Indeed, higher cardiorespiratory fitness levels have been associated with better performance on a relational memory task and greater hippocampal volumes in children [30], findings that have been recapitulated in adolescents [31, 32]. Together, these results suggest that the pervasive central benefits of PA on cognition span age groups.

Clinical studies demonstrate a positive relationship between PA and brain structure and function. A neuroanatomical study of persons aged 55 to 79 years demonstrated that age-related declines in cortical tissue density in the frontal, temporal, and parietal cortices were significantly reduced as a function of cardiovascular fitness [33], an interesting fact given that these areas underlie executive function and yet exhibit the greatest rate of age-related decline in humans [34]. Another study of elderly persons showed a direct correlation between increased levels of PA and improved cognition, with increased hippocampal volume seen after chronic exercise [35], supporting the idea that PA may prevent age-related anatomical and physiological deterioration in the brain [36, 37].

Bolstering the notion of PA's positive central effects are preclinical and clinical studies demonstrating neuroprotective and neuroplastic effects across a variety of neurodegenerative and neuropsychiatric diseases [36, 38–44]. A recent systematic analysis of 38 animal and human studies reported that PA attenuates Alzheimer-related neuropathology and positively affects hippocampal-mediated cognitive function, particularly when deployed early in the disease process [36]. Findings from another systematic review and meta-analysis demonstrate that PA is beneficial for people with Parkinson's disorder, specifically in areas of physical functioning, health-related quality of life, strength, balance, and gait speed [45]. Moreover, a recent review of clinical trials demonstrated that acute and chronic exercise generally increased levels of trophic factors in plasma and serum in persons with neurodegenerative conditions, including those with multiple sclerosis [46]. Also, PA has shown clear and consistent promise in promoting neuroplasticity in persons with mood disorders and, thereby, improving behavioral and neurobiological outcomes [38, 47], effects that extend to persons with posttraumatic stress disorder [48]. In persons with schizophrenia, evidence suggests that PA improves global cognition, working memory, social cognition, and attention [49]. A randomized controlled trial in persons with schizophrenia demonstrated that PA induced a 12% increase in hippocampal volume relative to nonexercisers [50]. While the dynamic cellular and molecular cascades that underlie the association between PA, cognition, and brain structure and function have yet to be elucidated fully, several modifiable mechanisms that alter neural plasticity have garnered increased attention recently, especially neurotrophic signaling, neurogenesis, inflammation, stress response, and antioxidant defense mechanisms [16]. Admittedly, an exhaustive review of all factors related to cognitive aging is beyond the scope of this article. Therefore, the reader is referred to the following excellent reviews for other factors that have been implicated in cognitive aging [51–59].

### 2.1. Neurotrophic Signaling

Neurotrophins are essential modulators of PA-induced neural plasticity. As one of the most widely distributed neurotrophins in the brain, BDNF plays a critical role in the maintenance, growth, and synaptic plasticity of neurons that underlie emotion and cognition [18, 60–62] and also modifies neuronal excitability [62, 63]. BDNF is centrally and peripherally upregulated [64–67] following acute and long-term PA [68, 69], changes that endure for days [70] and are prominent in the hippocampus [22]. While higher levels of training intensity are requisite for maximal effects [66, 71], both resistance [72] and aerobic [71] exercise can effectuate the increases in BDNF levels once sufficient intensity of PA is achieved.

Extending these studies to humans, it has been shown that moderate levels of PA mitigate cognitive decline in aging persons through putative mechanisms that involve BDNF. Laurin and colleagues demonstrated that PA levels were inversely correlated with the risk for cognitive impairment and all-cause dementia [73]. Lautenschlager and colleagues reported that persons with subjective memory impairments who were randomized to 6 months of aerobic exercise exhibited lower clinical dementia rating scores, increased delayed recall, and better outcomes on the cognitive subscale scores of the Alzheimer's Disease Assessment Scale relative to controls during an 18-month follow-up period [74]. Coelho and colleagues investigated the effects of acute aerobic exercise on BDNF levels in older persons with AD and found a significant correlation between BDNF levels and levels of PA [75], suggesting that long-term PA may persistently elevate BDNF levels and modulate cognitive function in older adults. The latter notion is important given that BDNF gene expression levels naturally decrease in age-related disorders such as AD [76]. Decrements in BDNF are problematic because retrograde transport of BDNF from the hippocampus to forebrain cholinergic neurons protects against neuronal damage and degeneration [66]. Moreover, the maintenance of basal BDNF levels is requisite for hippocampal neurogenesis [77]. Interestingly, while both PA and cognitive training improve cognitive function, only PA increases plasma BDNF levels in rodents, suggesting that an adequate level of PA is essential for BDNF-mediated plasticity [78]. Furthering this notion is work demonstrating that the blockade of BDNF on TrkB receptors reduced the positive effects of PA on synaptic plasticity [79].

Altogether, these results suggest that PA effectuates central neuroplastic adaptations via the optimization of BDNF levels. The ability of PA to enhance BDNF release and function in the synapse, to promote dendritic spine integrity, and to activate other cellular pathways that contribute to plasticity [80–83] is a cornerstone for homeostatic processes that maintain, repair, and reorganize circuits damaged during aging and disease.

### 2.2. Neurogenesis

The addition of new neurons to existing circuits through adult neurogenesis represents a unique form of synaptic plasticity. The majority of the neurons in the brain are formed in the womb. However, the brain maintains the ability to generate new neurons throughout life in certain regions (e.g., dentate gyrus and olfactory bulb) [84, 85]. Importantly, preclinical work suggests that PA increases adult neurogenesis, synaptic plasticity, and learning in the dentate gyrus of the hippocampus. Van Praag and colleagues demonstrated that voluntary wheel running simultaneously increased bromodeoxyuridine-positive cell numbers (precursor cell proliferation) and improved water maze performance (learning) [17]. Schmidt-Hieber and colleagues showed that newly born neurons in the hippocampus exhibit a lower excitability threshold and enhanced capabilities for synaptic plasticity [86], altering the rate by which new dentate granule cells are functionally integrated into hippocampal circuitry [87]. Eadie et al. demonstrated that long-term PA significantly increased total length and complexity of dendrites. Fascinatingly, they also demonstrated that long-term PA induced a more immature state of dentate granule cells [19], suggesting that PA reopens windows of plasticity. Stranahan et al. demonstrated that long-term voluntary wheel running in rats induced changes in spine density along with changes in arborization and spine morphology [20]. Others demonstrated that PA and cognitive stimulation exert differential effects on neurogenesis in rodents [88–91]. Whereas PA increases proliferation of neural precursor cells, cognitive stimulation promotes survival of the newly born cells. Thus, the absence of complex stimulation can block differentiation into mature neurons [92].

Translating the preclinical work to humans, clinical investigations using functional magnetic imaging have demonstrated that long-term aerobic exercise (3 months) increased blood volume in the dentate gyrus of the hippocampus and improved performance on the modified Rey Auditory Verbal Learning Test [93]. A randomized controlled study of healthy community-dwelling older adults demonstrated that those who participated in moderate aerobic exercise 3 times per week for 12 months showed a significant increase in size in the right and left hippocampus with concomitant improvements in spatial memory, a reversal that mitigated 1-2 years of age-related loss in hippocampal volume [94]. Encouragingly, increases in hippocampal size have been correlated with increases in spatial memory performance in both healthy adults [94] and persons with mild cognitive impairment [95]. The fact that PA upregulates neuronal proliferation and increases plasticity offers much hope for exploiting newly born neurons to maintain hippocampal volume in healthy and high-risk populations during aging [36, 96].

### 2.3. Inflammation

Long-term PA upregulates anti-inflammatory processes, an important finding given that chronic inflammation is mechanistically linked to cognitive impairment, mood disorders, cardiovascular diseases, and neurodegenerative disorders [22, 97]. Several studies have demonstrated that persons who regularly participate in PA have fewer viral and bacterial infections and a reduced incidence of systemic low-grade inflammation [98–102]. For instance, Kohut and colleagues studied the effects of PA on immune function and found that elderly individuals who participated in aerobic exercise (45 minutes per day, 3 days/week for 10 months) exhibited a reduction in plasma interleukin 6 (IL-6), interleukin 8 (IL-8), C-reactive protein (CRP), and tumor necrosis factor (TNF) levels [101]. A randomized control trial in sedentary elderly adults demonstrated that those who participated in a supervised exercise program (3 days/week for 6 months) showed improvement in their inflammatory profile [103]. Other studies suggest that the beneficial effects of long-term exercise on cognition may stem in part from anti-inflammatory factors, specifically IL-6 [104–107], IL-8 [108–110], CRP [111–113], and TNF [114–116]. These findings are in line with several recent reviews that found that long-term moderate intensity PA can exert anti-inflammatory and neuroprotective effects [117–122]. Moreover, a recent review explicated mechanisms that contribute to neuroinflammation-induced impairments in neurogenesis in several conditions (aging, Alzheimer's, traumatic brain injury, and stroke), underscoring the importance of therapeutics such as PA that target the interplay between multiple neuroplasticity substrates, not isolated factors per se [123]. Together, these studies offer hope that PA can be used to mitigate age-related changes in immune senescence and preserve cognitive function with aging.

### 2.4. Stress Response

The hypothalamic-pituitary-adrenal axis (HPA) is a neuroendocrine circuit that coordinates emotional, cognitive, autonomic, and neuroendocrine responses to acute and chronic stress. Acute deactivation and activation of the HPA effectuates various changes in brain activation patterns: significant deactivation occurs in the hippocampus, hypothalamus, medio-orbitofrontal cortex, and anterior cingulate cortex following stress [124], whereas significant activation occurs in the amygdala [125, 126]. These activation patterns likely reflect adaptations to help a person recognize and counteract similar stressors in the future [127]. Conversely, persistent activation of the HPA as a result of chronic stress can mediate long-term changes in the stress response including damage to keys areas of the brain (e.g., prefrontal cortex, paraventricular neurons, and hippocampus) [127]. It has been shown that persistently elevated levels of glucocorticoids are neurotoxic [128, 129]. Specifically, HPA dysregulation induces neuronal atrophy secondary to changes in neurochemistry, resilience, and plasticity in the hippocampus [130].

Activation of the HPA is induced by corticotropin-releasing hormone (CRH) in the paraventricular nucleus in response to a stressor challenge, which induces adrenocorticotropic hormone (ACTH) from the pituitary and, in turn, effectuates the release of glucocorticoids (cortisol in humans and corticosterone in rodents) from the adrenal glands [131]. Glucocorticoids then modulate the stress response along with metabolic, immunologic, and genetic functioning [132–134]. Notably, the release of cortisol following an HPA stress response occurs within the context of ongoing basal cortisol release. That is, cortisol is naturally secreted over a 24-hour period daily in the absence of stressors according to a diurnal cycle [135]. Notwithstanding, cortisol levels naturally vary in response to endogenous and exogenous factors (e.g., sleep wake cycle, exposure to light and dark, hormones, food consumption, and psychosocial variables) [136]. Thus, HPA function reflects an individual's basal diurnal secretion along with their response to ongoing endogenous and exogenous stress.

Negative feedback mechanisms tightly regulate the HPA response via mechanisms that involve high-affinity binding to mineralocorticoid receptors and low affinity glucocorticoid receptors [137]. Glucocorticoids “turn off” their own secretion by downregulating the release of hormones (CRH and ACTH), a response that then decreases mineralocorticoid and glucocorticoid receptor signaling and, in turn, downregulates the activity of the HPA to prestress baselines. Appropriate modulation of the HPA response appears paramount to brain health given several lines of evidence implicating stress-related hyperactivity and dysregulation of the HPA with age-related, neuropsychiatric, and neurodegenerative disorders [128, 133, 138–142].

Unfortunately, some evidence suggests that HPA changes may occur during the aging process. It has been shown that cortisol levels increase with age [143, 144] and diurnal slopes flatten [144–147]. Aging also engenders decreased glucocorticoid sensitivity and impaired negative feedback, changes that could prolong the stress response [148]. Finally, the HPA axis may become dysregulated in aging persons following exposure to chronic stress (e.g., health impairments, loss of function, and bereavement) [149]. With time, these changes may effectuate systemic changes that are deleterious to physical and cognitive health. Indeed, increased basal cortisol levels are associated with hippocampal-related memory impairments [150] and frailty [151], whereas lower levels of basal cortisol are associated with longevity [152].

Fortunately, a bevy of research suggests that long-term, voluntary PA mitigates an overactive stress response [153, 154]. Supporting this notion is evidence that exercise reduces the response to stressor challenge [155], an effect that may stem from exercise-induced fluctuations of glucocorticoid and mineralocorticoid receptor expression in the brain [155, 156]. The ability of PA to attenuate rises in cortisol levels may be especially important for preventing hippocampal atrophy [157–159] and for reversing cognitive deficits in the aging population [94, 160] given that hippocampal neurons exposed to persistently elevated glucocorticoids retract their dendrites and exhibit fewer dendritic spines [161]. Also, preclinical evidence suggests that the degree of dendritic branching in hippocampal neurons and overall number of dendritic spines increase with voluntary wheel running [19, 20, 162], potentially mitigating the effects of stress exposure. Together, this evidence suggests that PA may bolster physiological resilience by optimizing the stress response during aging.

### 2.5. Antioxidant Protection

Humans have a highly evolved antioxidant system designed to protect neurons from oxidative stress. By definition, oxidative stress is an imbalance between antioxidants and reactive oxygen species (ROS) (e.g., superoxide, hydrogen peroxide, and hydroxyl radical) [163]. Oxidative stress is widely deleterious in the central nervous system given that reactive oxygen species damage proteins, DNA, and lipids [164] and the fact that the brain has high metabolic demands and low antioxidant capacity [165, 166]. Notwithstanding, aerobic exercise decreases overall levels of ROS and increases adaptations to ROS-induced lipid peroxidation [167, 168]. These mechanisms stem in part from the ability of PA to increase antioxidant gene expression (e.g., superoxide dismutases and glutathione peroxidase) and, thereby, antioxidant enzymatic activities in the brain [167, 169]. Together, these studies suggest that long-term exercise optimizes redox homeostasis. Such is important for aging persons given that the kinase proteins that induce structural and functional changes in synapses require specific redox environments and that synaptic activity can be modulated via ROS levels.

## 3. Cognitive Engagement as a Component of Healthy Lifestyle

Convergent evidence suggests that engagement in mental activity also conveys neuroprotective and neuroplastic benefits during aging. Higher levels of education, a proxy for cognitive reserve, are associated with a reduced risk for cognitive impairment [170, 171], even in those with high-risk genetic backgrounds (e.g., apolipoprotein E4 carriers) [172, 173], possibly by increasing the threshold at which impairments become clinically manifest [174]. Another study demonstrates that higher education is protective against cognitive deficits in elderly individuals with white matter lesions [175]. Moreover, persons engaged in cognitively demanding occupational [176–179], leisure [180, 181], and social activities exhibit a reduced risk for cognitive decline with aging [13, 14, 176–180, 182–188]. Leisure activities that have demonstrated procognitive effects include reading, discussion groups, computer usage, participation in card and board games, solving puzzles, playing musical instruments, and learning a second language [180, 189–194]. Social activities that have demonstrated procognitive effects include traveling; attending theater, concerts, or art events; participating in social groups or pension organizations; socializing with family; and dancing [180, 191, 192, 194].

Underlying the effects of mental, leisure, and social engagement on cognition is a concept called “reserve.” According to the reserve hypothesis, impairments in cognition become manifest after a pool of brain and cognitive resources is depleted. Brain reserve refers to structural differences that increase tolerance to pathology, whereas cognitive reserve refers to variability in approach to task performance. The idea of brain reserve derives from studies showing that the occurrence of dementia is lower in persons with larger brain weights [195, 196] and that persons who engage in intellectually stimulating activities experience less hippocampal atrophy with aging [197]. Cognitive reserve suggests that a person can mitigate the effects of brain pathology by deploying pre-existing processing approaches or by deriving alternative strategies [198, 199]. By corollary, persons with decreased brain or cognitive reserve are more likely to exhibit clinical impairments with age- or disease-related insult given their fewer brain resources, whereas those with a higher reserve have more resources to rely upon following age- or disease-related insult, raising their threshold for clinical impairments.

Contemporary views of brain and cognitive reserve espouse more nuanced conceptualizations. Enriched environments infused with challenging activities are thought to effectuate the formation of new dendritic branches and synapses. These morphological changes then deepen the brain's capacity to resist insult while increasing augmentation of glial support cells, enhancement of the brain's capillary network, and the induction and incorporation of new neurons [200]. Indeed, preclinical work shows that stimulating environments increase neurogenesis [17, 201, 202] and upregulate BDNF [203–205], benefits that contribute to neural plasticity and extend to aging animals [206]. Enriched physical and social environments may provide short-lived mild to moderate stressors that induce locus coeruleus neurons to release noradrenaline and facilitate the formation and maintenance of adaptive memories [47], a process that could enhance adaptive structural changes in the brain (brain reserve) and cognitive and socioemotional learning (cognitive reserve). Supporting the latter notion is a multiplicity of studies showing that mental and socioemotional factors—including positive coping, optimism, sense of purpose, self-efficacy, and social support—are correlated with the stress response [207], are essential for the maintenance of high resilience [208–215], and are vital for mitigating age-related cognitive decline [216–218].

Another strategy that is garnering increased attention for enhancing brain and cognitive reserve is mindfulness meditation. A meta-analytic review (of 21 studies with approximately 300 participants) by Fox and colleagues examined the structural brain changes associated with mindfulness meditation and found that several brain regions consistently exhibited morphological differences in practitioners: the frontopolar cortex, sensory cortex, insula, anterior and mid-cingulate, hippocampus, and orbitofrontal cortex [219]. These areas are known to participate in awareness, attention, and emotional regulation, but are adversely affected in age-related disease and mood disorders [220, 221]. Tang and colleagues [222] reviewed a myriad of studies to determine the effects of mindfulness meditation on structural brain changes, functional activation, and neural connectivity. These authors reported that mindfulness meditation was associated with structural (in the prefrontal cortex, anterior and posterior cingulate, insula, hippocampus, and amygdala), functional activation (prefrontal cortex, anterior cingulate, amygdala, insula, and orbitofrontal cortex), and neuroplastic changes (anterior cingulate cortex and prefrontal cortex) in the brain of meditators versus controls [222]. While the underlying mechanisms that contribute to the structural, functional, and neuroplastic changes associated with mindfulness have yet to be elucidated fully, it seems plausible that neurogenesis, dendritic branching, and synaptogenesis may be involved in emotional and cognitive regions of the brain, particularly given that meditation reduces cortisol release following stress [223–225].

Correspondingly, it is also held that cognitive rehabilitative protocols may serve as a form of enriched environment and effectuate cognitive gains in the aging population. Approaches to cognitive rehabilitation involve exercises carefully designed to harness neuroplasticity. Investigating the effects of cognitive rehabilitation in healthy older adults and persons with mild cognitive impairment, a Cochrane review demonstrated that immediate and delayed verbal recall improved significantly following training as compared to a no-treatment control condition [226]. Extending these studies further, another review assessed the effect of cognitive interventions on activities of daily living, mood, quality of life, and metacognition in persons with mild cognitive impairment. The authors found that computerized cognitive interventions conferred benefits to mood compared to controls, whereas therapist-based and multimodal interventions had a greater impact on activities of daily living and metacognitive outcomes than control conditions [227]. The notion that computerized cognitive rehabilitation may convey positive cognitive effects during aging is intriguing given that (1) these techniques can be deployed in a relatively quick and cost-effective manner; (2) the training can be personalized; (3) the rehabilitation can be used to target vulnerable and underserved populations, that is, persons who are homebound, residents of nursing homes, and those without access to transportation; and (4) preliminary evidence suggests residual effects are retained long-term (5 years) [228].

## 4. Diet and Healthy Lifestyle

Food consumption is an intrinsically motivated behavior with the potential to modulate brain structure and function. Driving this behavior is energy demand: whereas the brain comprises 2% of total body weight, it consumes 20% of the total energy derived from nutrients [229]. The exorbitant demand for energy derives from the requisite needs of neurons to maintain ionic gradients across their membranes to facilitate neurotransmission via oxidative metabolism. Accordingly, neurons are extremely sensitive to mitochondrial dysfunction and oxidative stress [166, 230, 231].

The centrality of feeding behavior for survivability makes it seem plausible that optimized food consumption represents a means to impact brain function positively. This putative effect stems in part from the ability of dietary factors to modulate synaptic plasticity by altering neurogenesis, inflammation, antioxidant defense mechanisms, neurotrophin levels, and energy metabolism [232], mechanisms similar to those induced by long-term PA [16]. For example, preclinical studies suggest that increased consumption of dietary fructose in the presence of an omega-3 fatty acid deficiency adversely affects learning and memory [233] by altering the function of molecules that are important in mitochondrial bioenergetics [234] in key brain regions such as the hippocampus [235]. Parallel evidence demonstrates that nutritional content, along with the level and frequency of food intake, effectuates changes in energy metabolism and neuroplasticity [229]. Population-based studies suggest that diets rich in polyphenols promote better performance in several cognitive abilities in a dose-dependent manner [9] and lower the risk of cognitive decline [10, 11] in older persons. Accordingly, it is increasingly held that bioactive substances in food represent a novel target for lifestyle interventions that may promote healthy brain aging and preserve cognitive function, especially in aging adults at risk for nutritional deficits [236]. Given that dietary modifications are considered by many to be safer and more easily integrated into lifestyle changes than conventional pharmacotherapeutics, several bioactive substances that have received intense investigation are reviewed below in brief.

Polyphenols (e.g., phenolic acids, stilbenes, lignans, flavonols, and anthocyanidins) comprise a class of approximately 8000 compounds with antioxidant properties. These compounds are found in fruits, vegetables, tea, wine, juices, plants, and some herbs. Whereas polyphenols are not considered “essential nutrients,” convergent evidence does suggest that these factors can mitigate risk for neurodegenerative diseases, age-related cognitive decline, and oxidative stress [12, 237–245] via mechanisms involving the maintenance of metabolic homeostasis [241, 246] and the promotion of synaptic plasticity [241, 247]. Several dietary choices of polyphenols with putative neuroprotective [232], neuroplastic [248], neurogenic [249–251], and anti-inflammatory effects [252] have been explored, with a particular emphasis on curcumin, catechins, resveratrol, and omega-3 fatty acids.

### 4.1. Curcumin

As a plant-based diarylheptanoid produced by the plant turmeric, curcumin is a component of yellow curry spice. This bright-yellow pigment was first isolated more than a century ago and has been used extensively in Indian medicine. Historically, it has been deployed to mitigation inflammation [253, 254], oxidative damage [255], and amyloid build-up [256, 257]. The antioxidant capabilities of curcumin appear to stem from its unique structure that can donate H-atoms or transfer electrons from two phenolic sites, allowing it to scavenge free radicals easily. More recently, curcumin has garnered attention for its effects on neuroplasticity and its ability to ameliorate processes involved in brain aging and neurodegeneration.

Preclinical investigations show that dietary supplementation of curcumin 3 weeks prior to [258] and after [259] experimentally induced traumatic brain injury partially ameliorate the consequence of injury on markers of synaptic plasticity (e.g., BDNF and cAMP response element-binding protein), mechanisms that may partly involve the restoration of energy homeostasis [258–260] and facilitation of neurogenesis in the dentate gyrus of the hippocampus [261]. Also, curcumin may prevent secondary sequelae following brain injury by inhibiting the formation of oligomers and fibrils and the aggregation of amyloid proteins [262–264]. Interestingly, curcumin appears to cross the blood-brain barrier. Curcumin injected into the tail vein of rodents altered plaque formation in a model of AD [265]. A recent meta-analysis and systematic review of eight preclinical studies demonstrated that curcumin significantly improved neurological function in the central nervous system, an effect that was proportional to dosage [266].

Recently, preclinical studies have focused on the effects of curcumin administration on aging. One recent study has demonstrated that curcumin rescued age-related loss of hippocampal synapse input specificity of LTP by favoring N-methyl-D-aspartic acid receptor activity [267]. Also, curcumin and its metabolite, tetrahydrocurcumin, increased the mean lifespan of at least three model organisms [268] and modulated the expression of aging genes in some models [269].

Extending these studies to humans, a large population-based study of elderly nondemented Asians investigated the association between curry consumption and cognitive function, finding that persons who frequently consumed curry scored significantly better on the Mini-Mental State Examination relative to those who infrequently consumed curry [270]. Another 6-month randomized, placebo-controlled, double-blind, clinical study of curcumin in persons with progressive cognitive decline and memory found increased serum amyloid beta-40, but not improvements on the Mini-Mental State Examination [271]. Cox and colleagues investigated the acute, chronic, and acute-on-chronic effects of a curcumin formulation (400 mg) on cognitive function, mood, and blood biomarkers in healthy older adults. They found that curcumin significantly improved (1) performance in attention and working memory 1 hour following administration as compared with placebo, (2) working memory and mood following 4 weeks of treatment, (3) alertness and contentedness 1 hour and 3 hours after a single dose following chronic treatment, and (4) LDL cholesterol via reduced total concentration [272]. Daily and colleagues examined the efficacy of curcumin for alleviating the symptoms of arthritis and found supportive treatment evidence for turmeric extract (about 1000 mg/day of curcumin) [273], suggesting a translational avenue for its anti-inflammatory effects. Derosa and colleagues evaluated the efficacy of curcuminoid supplementation on circulating concentrations of IL-6 in randomized controlled trials and reported a significant effect of curcumin in lowering circulating IL-6 concentrations, an effect that was more evident in patients with greater systemic inflammation [274]. A systematic review and meta-analysis of randomized controlled trials evaluated the efficacy of curcumin supplementation on circulating levels of TNF-α and reported a significant effect of curcumin in lowering circulating TNF-α concentration [275]. The ability of curcumin to mitigate chronic inflammatory processes is important because chronic inflammation dysregulates neurotransmission and trophic factor signaling and disrupts the processes of neurogenesis and neuroplasticity [276–279]. Moreover, chronic inflammatory processes can contribute to glutamate-mediated excitotoxicity [279] and loss and dysfunction of glial cells [280–282].

To date, the results from preclinical research suggest that curcumin may benefit the brain and cognitive function during aging, but the level of evidence is still weak. One of the main limitations with curcumin studies and interventions is related to its limited bioavailability, a factor that could be addressed by chemical modification, conjugation with lipophilic compounds or coadministration with other compounds. No clinical trials to date provide conclusive evidence of the efficacy of long-term curcumin consumption for preventing or treating cognitive decline with aging. More studies are needed to explore the effects of this factor in persons with different genetic backgrounds and at different states of health and wellness.

### 4.2. Catechin Polyphenols

Found naturally in teas, catechin polyphenols are potent bioactive compounds with antioxidant [283, 284] and anti-inflammatory properties [285, 286]. Their ability to donate hydrogens and scavenge reactive oxygen and nitrogen species underlies their antioxidant capabilities [283, 284]. Among the catechins found in tea, (−)epigallocatechin-3-gallate (EGCG) is a major constituent and therapeutic agent. EGCG has been shown to have neuroprotective functions that include antioxidant, iron chelating, and anti-inflammatory properties [287, 288]. Also, EGCG promotes amyloid precursor protein processing via the nontoxic amyloid precursor pathway [289] to reduce amyloid-beta pathology [290]. EGCG also appears to modulate cell survival genes [291].

Emerging preclinical and clinical evidence has suggested that EGCG modulates mechanisms involved in learning and cognitive decline. EGCG facilitated glutamate release by enhancing Ca^2+^ entry through voltage-dependent Ca^2+^ channels in isolated nerve terminals from rat cerebral cortex, a process linked to protein kinase C (PKC) activation [289, 291, 292]. This ability is important because increased release of glutamate in the brain has been shown to be a proxy for learning and memory [293, 294]. EGCG also affected synaptic plasticity as high-frequency stimulation-evoked LTP was enhanced following preincubation of hippocampal slices with EGCG [295]. Another study has demonstrated that the application of EGCG modulated synaptic transmission and produced a dose-dependent improvement in the induction of LTP in the rat in vivo [296]. Moreover, long-term administration of green tea catechins to rats improved their reference and working memory-related learning ability and decreased reactive oxygen species concentrations in the hippocampus [297]. These results are not surprising given the relationship of EGCG to neurogenesis and BDNF: oral administration of EGCG enhances cell proliferation and increases the number of progenitor cells in the hippocampus of rodents [250, 251]. Submicromolar concentrations of EGCG (<0.1 *μ*g/ml) of unfractionated green tea and low concentrations (<0.5 *μ*M) of EGCG potentiated the neuritogenic ability of low-concentration BDNF [298].

Parallel study has investigated the effects of catechins in humans. A large-scale study of middle-aged adults investigated the long-term association between polyphenol intake and cognitive performance, finding that catechins were positively associated with language and verbal memory [299]. A study of community-living Chinese adults aged 55 years or older demonstrated that consumption of black and oolong tea was associated with lower risks of cognitive impairment and decline after a 1- to 2-year follow-up [300]. A cross-sectional study of community-dwelling Japanese adults aged 70 years or older examined the association between green tea consumption and cognitive function, finding that higher consumption of green tea was associated with a lower prevalence of cognitive impairment as assessed by the Mini-Mental State Examination [301]. In a small interventional study in healthy volunteers, increased brain activity on functional magnetic resonance imaging in the dorsolateral prefrontal cortex, a proxy for memory processing, was reported in a dose-dependent manner following administration of green tea [302]. The effects of green tea consumption on the brain activity of healthy volunteers were measured using simplified EEG during passive activity in another study, with findings demonstrating significantly increased theta waves between 30 minutes and 1-hour postconsumption, suggesting a role for enhancing cognitive function [303].

### 4.3. Resveratrol

As a plant-based stilbene found in grapes, wine, and peanuts, resveratrol possesses significant free radical scavenging capabilities [304] given its three OH groups in positions 3, 4, and 5; aromatic rings; and a double bond in the molecule. Recently, it has garnered increased attention amidst reports of its neuroprotective and antiamyloid properties [305, 306] in rodents through mechanisms that likely involve oxidative stress [306], energy homeostasis [307], and neural plasticity [308, 309]. Bolstering this notion are cell culture studies that demonstrated that resveratrol reduced amyloid beta accumulation, ROS, and apoptosis [310] via modulation of nuclear factor-kB and Sirtuin 1 pathways [310–312]. Some preclinical studies suggest that resveratrol extends the lifespan [310, 313, 314]. For example, resveratrol increased cell survival by stimulating Sirtuin 2, a change that increased DNA stability, slowed aging, and extended the lifespan by 70% in yeast models [315]. Resveratrol added to the food of seasonal fish in early adulthood induced a dose-dependent increase of median and maximum lifespan [313]. Dietary consumption of resveratrol enhanced proliferative states in neuronal stem cells in the rat hippocampus [316]. Several parallel preclinical studies have demonstrated that resveratrol attenuated stress-induced learning deficits, depressive symptoms, and hippocampal degeneration by mechanisms that involved the restoration of BDNF [308, 309, 317–320]. Altogether, this preclinical data provides evidence that resveratrol treatment may be efficacious for improving mood and cognitive function.

Extending this line of investigation to humans, one small-scale, randomized, placebo-controlled, double-blind trial with Concord grape juice supplementation for 12 weeks demonstrated that older adults with memory decline but not dementia significantly improved in a measure of verbal learning [321]. Also, a double-blind, placebo-controlled study tested whether supplementation with resveratrol enhanced memory performance in older adults, finding that administration of 200 mg of resveratrol daily with 320 mg quercetin for six months duration in healthy older adults (50–80 years) effectuated greater hippocampal activity at rest (as assessed by functional magnetic resonance imaging) and improved memory performance [322]. Notwithstanding, low bioavailability of resveratrol is a major drawback [323]. Therefore, methods to enhance bioavailability (nanosized particles and oral lozenges) are being investigated [324–326].

### 4.4. Omega-3 Fatty Acids

Whereas transfats have deleterious effects in the brain, omega-3 fatty acids (found in oily fish such as salmon, mackerel, herring, anchovies, menhaden, and sardines) have neuroprotective effects. Omega-3 fatty acids [e.g., *α*-linolenic acid, eicosapentaenoic acid, and docosahexaenoic acid (DHA)] are polyunsaturated fatty acids that are vitally involved in neuronal physiology. Among the omega-3 fatty acid family members is DHA, one of the most important because of its role in maintaining the structural balance of cell membranes, its ability to mediate phospholipid signal transduction at the synapse, and its ability to modulate enzymatic activity [327, 328]. Also, DHA stabilizes molecular mechanisms important for mitochondrial function [329], brain glucose utilization [330], and oxidative stress [331]. Dietary DHA also contributes to epigenetic changes that confer resilience to metabolic perturbations [332]. Notably, humans are reliant on consumption of dietary DHA from oily fish since the body is inefficient at synthesizing it. Clinical evidence suggests that dietary deficiencies can have adverse cognitive effects [333], yet one study demonstrated that less than half of women consume the recommended dietary allowance [334, 335], a trend that can be reversed with supplementation. Preclinical studies show that dietary restrictions in omega-3 fatty acids are associated with reductions in neuronal size and neurotrophin levels [336], whereas dietary supplementation reverses age-related impairments in LTP and depolarization-induced glutamate transmitter release [337], effectuates increased levels of hippocampal neurotrophin levels [331], and upregulates genes that are important for maintaining synaptic function and plasticity [338].

A number of studies on omega-3 fatty acids have been extended to humans. Epidemiological studies demonstrate that high intake of fish rich in polyunsaturated fatty acids is associated with positive cognitive function. Results from the Rotterdam study demonstrate that high fish intake is inversely associated with incident dementia at baseline and at 2-year follow-up [339]. Elderly persons in the PAQUID cognitive and functional aging study who ate fish or seafood at least once a week exhibited a significantly lower risk of developing dementia in the 7-year follow-up period [340]. Similarly, community-dwelling elders in the Chicago Health and Aging Project who were in the upper quintile for consumption of saturated fat had a twofold increased risk for AD as compared to persons in the lowest quintile [341], suggesting that high intake of unsaturated, unhydrogenated fats may be protective against AD. Another study investigated whether omega-3 fatty acid intake is correlated with gray matter volume in brain structures associated with emotional circuitry, finding positive associations between reported dietary omega-3 intake and gray matter volume in the subgenual anterior cingulate cortex, right hippocampus, and right amygdala, intimating a mechanism by which omega-3 fatty acid intake may mediate memory, mood, and affect regulation [342]. Other study demonstrated that weekly consumption of baked or broiled fish is positively associated with increases in gray matter volumes in the hippocampus, precuneus, posterior cingulate, and orbitofrontal cortex [343]. Moreover, adults with subjective memory impairment who were administered fish oil (eicosapentaenoic acid + DHA) for 24 weeks in a randomized, double-blind, placebo-controlled study exhibited increased cortical blood oxygen level-dependent activity in the right posterior cingulate and left superior frontal regions during a memory task as well as enhanced overall working memory performance [344], results that mirror earlier results of the Framingham Heart Study wherein DHA levels in the top quartile were associated with a 47% lower risk of all-cause dementia [345]. Another study investigated the effects of DHA and arachidonic acid (240 mg/day of DHA and arachidonic acid) on cognition in amnesic patients and found that DHA supplementation improved cognitive dysfunction secondary to aging and organic brain pathology [346]. Finally, a recent study demonstrated that higher-fasting plasma levels of omega-3 polyunsaturated fatty acids correlated with larger gray matter volume within the left rostral anterior cingulate cortex, a characteristic that partially mediated the relationship between cognitive flexibility in at-risk (apolipoprotein E4 carriers) older adults [347].

Admittedly, a National Institute of Health State of the Science Conference panel previously concluded that there is insufficient evidence to recommend omega-3 fatty acids for age-related cognitive decline. Notwithstanding, there are ongoing clinical trials designed to elucidate efficacy, trials that may be chiefly beneficial for persons in the lower quartile of omega-3 consumption or in at-risk groups for cognitive decline [348].

### 4.5. Caloric Restriction and Intermittent Fasting

In the context of adequate consumption of nutrients, caloric restriction conveys lifespan and healthspan benefits, including preservation of cognitive function. Convergent evidence suggests that a reduction of caloric intake by 20–40% extends the lifespan of organisms throughout phylogeny [349]. Population studies in Danish and Norwegian men and women revealed that involuntary caloric restriction without malnutrition for periods of 2–4 years reduced overall mortality rates [350]. Moreover, it has been shown that centenarians from Okinawa consumed 17% fewer calories than average Japanese adults, and they consumed 40% fewer calories than American adults [333]. A recent review by Most and colleagues detailed the positive health benefits demonstrated from several recent randomized trials, reporting that caloric restriction in humans effectuate some of the same metabolic and molecular adaptations seen in preclinical models of longevity [351]. Finally, a 30% reduction in calories for 3 months has been associated with a 20% improvement in verbal memory in healthy elderly adults [352].

The mechanisms underlying caloric restriction appear to be multifold. Caloric restriction has been shown to increase cellular repair of DNA [353], reduce oxidative stress [354], improve the metabolism of glucose [355], and optimize immune [356] and neuroendocrine function [357, 358]. Moreover, caloric restriction counteracts age-related alterations in the expression of genes related to synaptic transmission [359]. For example, caloric restriction increases the expression of BDNF, TrkB, and NR2B subunits of NMDA receptors [359, 360] to mitigate age-related decrements in the hippocampus [361, 362]. Similarly, intermittent fasting exerts neuroprotective effects. It has been shown that synaptic resilience and function [363], levels of stress protein chaperones [364, 365], and neurotrophic factors [364] are increased following intermittent fasting, effects that may be particularly beneficial during times of injury [366].

## 5. Conclusions and Future Directions

Finding an effective treatment for age-related cognitive decline represents an unmet goal. However, considerable progress has been made in better understanding how PA and diet modulate common neuroplasticity substrates (neurotrophic signaling, neurogenesis, inflammation, stress response, and antioxidant defense mechanisms) in the brain [16]. Accordingly, this study highlights the importance of lifestyle modification for protecting cognitive function and brain health during aging and advocates for higher levels of PA and consumption of healthy foods to optimize neural plasticity. Once plasticity has been primed, cognitive training and rehabilitation can be used to facilitate the reorganization and proper function of cognitive circuits (to enhance brain reserve) and practice processing strategies and skills that translate to daily living (cognitive reserve). The deployment of techniques to optimize lifestyle are critical given the expanding size of the aging population juxtaposed with evidence that 97% of adults nationwide fail to exhibit healthy lifestyle characteristics [367]. Undoubtedly, the success of healthy lifestyle campaigns will require more emphasis on midlife, long-term, preventive approaches—with the goal of promoting positive health habits that delay progression and overt cognitive decline. Necessarily, these approaches should be paralleled by research that aims to disentangle the effects of lifestyle habits at different points along the aging and disease continuum.

Admittedly, large-scale, well-conducted, randomized controlled trials with PA, mental engagement, and dietary intake are only beginning to emerge. Undoubtedly, there is a need for future research in human populations that are well standardized and stratified in relation to genetic backgrounds, age, sex, and disease severity and duration. This research is needed to better understand the optimum mode, intensity, and duration of PA according to biologically distinct subgroups. Moreover, future studies will need to disentangle the individual and common pathways that exist between PA, mental activity, diet, social factors, and cognitive aging, particularly given evidence that various components may exert additive protection against cognitive decline [188]. In the area of cognitive rehabilitation, there remains a need to derive protocols whose outcomes reify as generalized, functional improvement in real-world environments [368]. While doing so, methodological standards will necessarily have to be considered more fully. It is known that noradrenergic function is essential for learning and memory [47] and that optimization of noradrenergic function (via PA or pharmacotherapeutics) during aging and disease [16, 47] may likely optimize learning and memory in certain populations (e.g., Down syndrome, Alzheimer's, and persons with mild cognitive impairment). Future studies should take into account the effects of noradrenergic function on cognitive training and rehabilitation outcomes in aging populations. Similarly, the duration of cognitive training and rehabilitation should be considered more fully. It is known that the effects of PA takes several weeks to reify at the behavioral level, a reflection of mechanisms that likely involve BDNF, neurogenesis, and the optimization of neurotransmitter levels [16]. Thus, it seems likely that the deployment of PA prior to cognitive training and rehabilitation could be used to normalize these factors to enhance outcomes, a notion that awaits further study. Finally, a greater understanding of antioxidant status in regard to plasma and brain bioavailability is needed, as are studies that disentangle dose-response effects, safety, tolerability, efficacy, and interactions with other dietary factors. The latter studies are imperative as it seems likely that the effects of nutrients in the brain are the product of a mélange of metabolites and interacting factors, not isolated factors per se. Together, these future efforts will help to ensure that research at the frontiers of cognitive neuroscience will provide a personalized approach to intervention during states of health, disease, and aging.

In the interim, the Alzheimer's Association and the AARP have launched public health initiatives that aim to foster a greater awareness of strategies that can be deployed to optimize cognitive function during aging. The initiative of the Alzheimer's Association is called Maintain Your Brain and promotes brain-centered healthy lifestyle choices (e.g., maintaining physical, mental, and social activity levels while concomitantly consuming a low-fat diet rich in antioxidants) [369]. Similarly, the AARP initiative, called Staying Sharp, encourages aging individuals to engage in a lifetime of learning and provides strategies to augment memory [370]. While neither program has been evaluated long-term, preliminary results from a two-year, multidomain, randomized, controlled study designed to prevent cognitive impairment are promising. The intervention consisted of PA, cognitive training, nutritional guidance, and social activities along with the management of vascular risk factors. The control group received regular health advice. After 2 years, a comprehensive neuropsychological test battery revealed a significant beneficial intervention effect on overall cognitive performance, including the domains of memory, executive function, and psychomotor speed. This novel study demonstrates the possibility of preventing cognitive decline using a multidomain intervention among older at-risk individuals [371]. It also highlights the importance in convincing patients of the value of a healthy lifestyle while concomitantly underscoring the importance of preventive public health policy.

## Abbreviations

AARP: American Association of Retired Persons
ACTH: Adrenocorticotropic hormone
AD: Alzheimer's disease
BDNF: Brain-derived neurotrophic factor
CRH: Corticotropin-releasing hormone
CRP: C-reactive protein
DHA: Docosahexaenoic acid
EGCG: (−)epigallocatechin-3-gallate
HPA: Hypothalamic-pituitary-adrenal axis
IL: Interleukin
LTP: Long-term potentiation
PA: Physical activity
ROS: Reactive oxygen species
TNF: Tumor necrosis factor.
